# Temporal Divergence of True and False Alarms in Pediatric Nocturnal Seizure Monitoring

**DOI:** 10.1002/acn3.70530

**Published:** 2026-09-29

**Authors:** Mohammad Shahbakhti, Anemoon T. Bosch, Job van der Palen, Franciscus S. Leijten, Johannes P. van Dijk, Richard H. C. Lazeron, Roland D. Thijs

**Affiliations:** ^1^ Stichting Epilepsie Instellingen Nederland (SEIN) Heemstede the Netherlands; ^2^ Department of Neurology Leiden University Medical Centre (LUMC) Leiden the Netherlands; ^3^ Department of Epidemiology Medisch Spectrum Twente Enschede the Netherlands; ^4^ Section Cognition, Data and Education, Faculty of Behavioural, Management and Social Sciences University of Twente Enschede the Netherlands; ^5^ Brain Center, Department of Neurology University Medical Center Utrecht Utrecht the Netherlands; ^6^ Academic Center of Epileptology Kempenhaeghe Heeze the Netherlands; ^7^ Faculty of Electrical Engineering Eindhoven University of Technology Eindhoven the Netherlands; ^8^ Department of Orthodontics Ulm University Ulm Germany; ^9^ UCL Queen Square Institute of Neurology London UK

**Keywords:** alarm fatigue, caregiver burden, children, epilepsy, wearable

## Abstract

Seizure detection devices can improve the safety of children with epilepsy, yet high false alarms limit their clinical applicability. Here, we investigated whether specific time periods are disproportionately prone to false alarms. We analyzed 2876 NightWatch alarms occurring between 19:00 and 09:00 in 65 children (ages 3–16, 48% female). Temporal distributions differed significantly between true and false alarms (*p* < 0.001), with a significant divergence in skewness (*p* = 0.001); notably, 30% of false alarms occurred between 06:00 and 09:00. This suggests NightWatch performance is adversely affected by wakefulness and morning arousal. Our findings support exploring time‐ or state‐dependent detection thresholds to reduce false alarms.

**Trial Registration:** The PROMISE (NCT03909984) and LICSENSE (NTR4115/NR395) trials were registered at the Dutch Trial Registry.

## Graphical abstract

The early‐morning surge in false seizure alarms supports exploring time‐ or state‐dependent detection thresholds to reduce false detections.

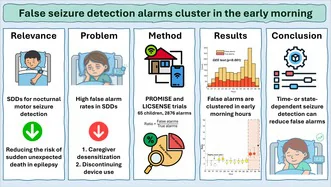

## 1 Introduction

Tonic–clonic seizures are associated with severe acute complications and remain the primary risk factor for sudden unexpected death in epilepsy, particularly when nocturnal [1, 2]. To mitigate these risks and provide peace of mind to caregivers, seizure detection devices (SDDs) were developed to detect nocturnal major motor seizures and alert caregivers. The practical utility of SDDs is, however, frequently undermined by high false alarm rates [3, 4]. This may trigger alarm fatigue—a phenomenon where caregivers become desensitized to alerts, potentially leading them to ignore genuine emergencies or discontinue device use altogether [5, 6, 7].

Despite the impact of high false alarms on SDD applicability, it remains unclear whether false alarms occur randomly or follow specific temporal patterns. Current detection algorithms generally operate under a static sensitivity model, assuming uniform physiological conditions throughout the night [8, 9, 10, 11]. If false alarms are disproportionately concentrated during specific intervals, this “one‐size‐fits‐all” approach may be fundamentally flawed. We therefore questioned whether the temporal pattern of true (nocturnal major motor seizure) versus false alarms differ and, if so, whether these patterns could help identify specific high risk windows for false alarms.

## 2 Materials and Methods

### 2.1 Data

We combined recordings from two prospective clinical trials: PROMISE (NCT03909984) [12] and LICSENSE (NTR4115/NR395) [13]. Both were registered at the Dutch Trial Registry and approved by the Medical Research Ethics Committee of the University Medical Centre Utrecht; written informed consent was obtained from the participants or assent from parents or legal guardians. In both trials, we validated the NightWatch (NW) system, a wearable armband that triggers alarms for nocturnal major motor seizures based on movement and heart rate. From the LICSENSE trial, we included only children whose alarms were generated by the most recent NW detection algorithm. Our final cohort comprised 65 children (ages 3–16; 48% female) recruited from three specialized Dutch epilepsy centers: SEIN, Kempenhaeghe, and University Medical Centre Utrecht. Children met the eligibility criteria by having a baseline frequency of at least one nocturnal major motor seizure per week.

Children wore the NW device on their upper arm—prioritizing the side with more prominent motor activity—for a median of 47 nights (interquartile range: 22–60). These sessions were conducted in the children's home or a residential care unit, with continuous video‐monitoring serving as the gold standard. The system transmitted wireless alerts to a caregiver station upon detection of suspected seizure activity. The NW specifically targets major motor seizures, a category encompassing generalized tonic–clonic, focal‐to‐bilateral tonic–clonic, generalized tonic seizures lasting over 30 s, focal onset clonic, generalized onset, and “tonic–clonic–like” bilateral events without a classic focal‐to‐bilateral pattern.

We labeled an alarm as true if a major motor seizure was identified within a 6‐min window (3 min before or after the trigger). Conversely, all alarms that did not meet these criteria, including minor seizures, were labeled as false.

### 2.2 Methods

We extracted timestamps for all true and false alarms and categorized them by their clock hour of occurrence. To prevent alarm clustering, we excluded any subsequent alarms occurring within 3 min of an initial detection from the analysis. We also restricted our analysis to a 14‐h nocturnal window (19:00–09:00) to encompass the typical pediatric nocturnal period while excluding daytime naps and testing periods. Then, we aggregated alarms into 1‐h time bins.

To determine whether false alarms exhibited a different temporal pattern than true alarms, we used generalized estimating equations (GEE). Each alarm represented one observation, with alarm type (true or false) as the binary outcome and clock hour as the predictor. The GEE model used a binomial distribution with a logit link function and an exchangeable working correlation structure based on child identification numbers to account for repeated observations within individuals. This population‐averaged approach estimates cohort‐level temporal effects while accounting for within‐child correlation, thereby preventing participants with high alarm frequencies from disproportionately influencing the results.

To account for differences in monitoring time due to varying bedtimes and wake‐up times among children, we normalized hourly alarm counts by the cumulative device‐hours within each clock‐hour bin, yielding time‐dependent alarm rates independent of variations in total device usage. We also compared the skewness of the true and false alarm distributions using a permutation test to determine whether either distribution exhibited greater temporal asymmetry across the 14‐h monitoring period.

We quantified the clinical impact of false alarms by calculating an hourly alarm ratio, defined as the ratio of false to true alarms within each hour. To assess the precision of these ratios, we also calculated 95% confidence intervals (CIs). Next, we normalized these hourly ratios against the nightly mean, calculated as the total number of false alarms divided by the total number of true alarms across the entire 14‐h monitoring period.

## 3 Results

### 3.1 Alarm Selection, Device Usage, and Active Child Counts

Figure 1a displays the step‐by‐step procedure for alarm selection. Across the 65 included children, a total of 3566 NW alarms occurred. Of these, we excluded 421 alarms due to insufficient video data, leaving 3145 annotated alarms. We then merged consecutive alarms within a 3‐min window to prevent clustering, resulting in 2950 unique alarm events. By restricting our analysis to the 14‐h nocturnal monitoring window (19:00–09:00), we excluded a further 74 alarms, yielding a final dataset of 2876 analyzed alarms, of which 742 (25.8%) were true. Finally, we aggregated the alarms into 1‐h bins. Figure 1b provides a unified temporal map of our final data, overlaying the raw hourly alarm counts. Figure 1c displays the subject‐level distribution, showing the number of unique children that contributed to alarms within each hourly interval, and Figure 1d displays the baseline device usage across the 14‐h window.

**FIGURE 1:**
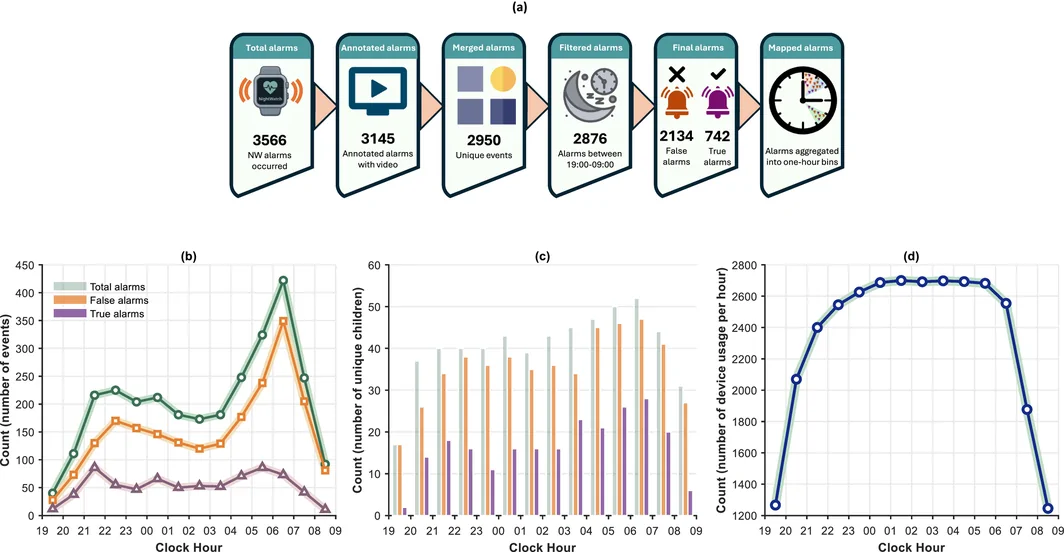
Overview of the alarm selection pipeline and hourly distribution profiles. (a) Flowchart detailing the sequential inclusion and filtering steps for the NW alarms, (b) temporal trends of the absolute volume (raw event count) of total, false, and true alarms aggregated into 1‐h bins across the 14‐h nocturnal window (19:00–09:00), (c) child‐level distribution displaying the absolute number of unique children contributing to total, false, and true alarms during each clock hour interval, and (d) baseline system exposure profile tracking the cumulative total device usage hours across the cohort for each hourly monitoring interval.

### 3.2 Comparison of Temporal Distribution of True and False Alarms

The temporal occurrence of alarms differed significantly between true and false alarms (GEE, p<0.001). True alarms were distributed throughout the nocturnal period with moderate peaks at 21:00–22:00 and 05:00–06:00. In contrast, false alarms were heavily skewed toward the morning transition (Figure 2a). A permutation test confirmed a significant divergence in skewness between the two distributions (true alarms: −0.1164; false alarms: −0.3373, bootstrap *p* = 0.001), displaying a robust standardized effect size (4.12 standard deviations from the null distribution). This statistically validates that the frequency of false alarms shifts progressively toward the end of the monitoring window, with 30.2% of false alarms concentrated within the 06:00–09:00 interval. Compared to a uniform distribution baseline—where any 3‐h block within our 14‐h monitoring window would be expected to contain only 21.4% of events—this morning cluster represents a relative enrichment of over 40% above the expected baseline, demonstrating a disproportionate concentration of system errors during waking transitions. The empirical cumulative distribution functions of alarms highlight this divergence (Figure 2b); while true alarms accumulated steadily, false alarms remained low during the early night before rising sharply after 06:00.

**FIGURE 2:**
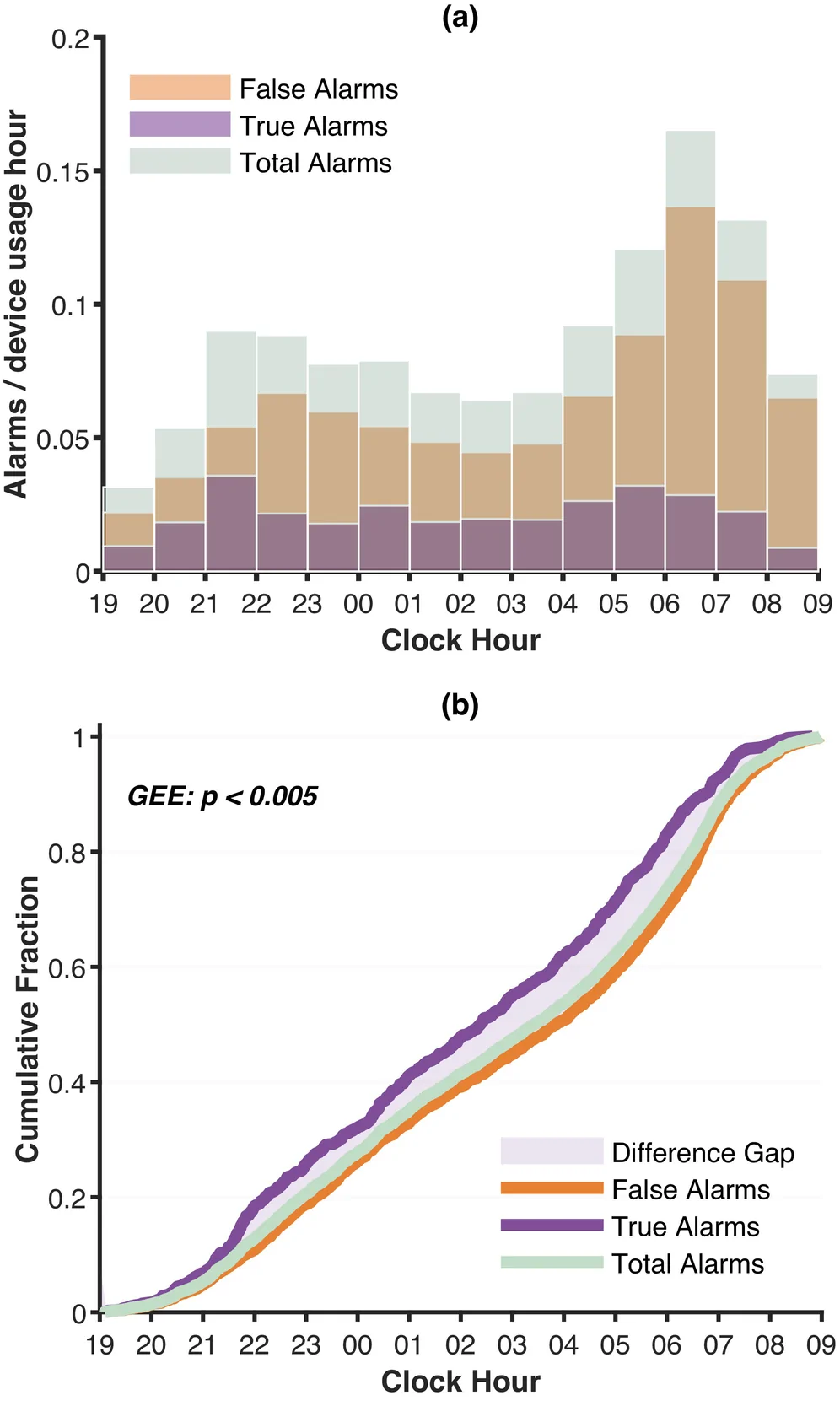
(a) Histogram showing the normalized hourly frequency of detected true, false, and total alarms relative to total device usage hours across the 14‐h window (19:00–09:00). (b) Cumulative fraction of alarms over the monitoring period; the vertical gap between curves illustrates a significant temporal divergence driven by a disproportionate accumulation of false alarms heavily skewed toward the morning transition window (06:00–09:00).

Temporal variations in true and false alarms caused the false‐to‐true alarm ratios to fluctuate significantly relative to the overall nightly mean (Figure 3). At the 20:00–21:00 and 21:00–22:00 intervals, the normalized ratios were 0.67 (95% CI [0.45, 0.97]) and 0.52 (95% CI [0.40, 0.68]), respectively. In contrast, the normalized ratios rose sharply during the morning intervals; between 06:00 and 09:00, they were 1.66 (95% CI [1.34, 2.13]), 1.70 (95% CI [1.24, 2.38]), and 2.56 (95% CI [1.51, 5.27]), respectively.

**FIGURE 3:**
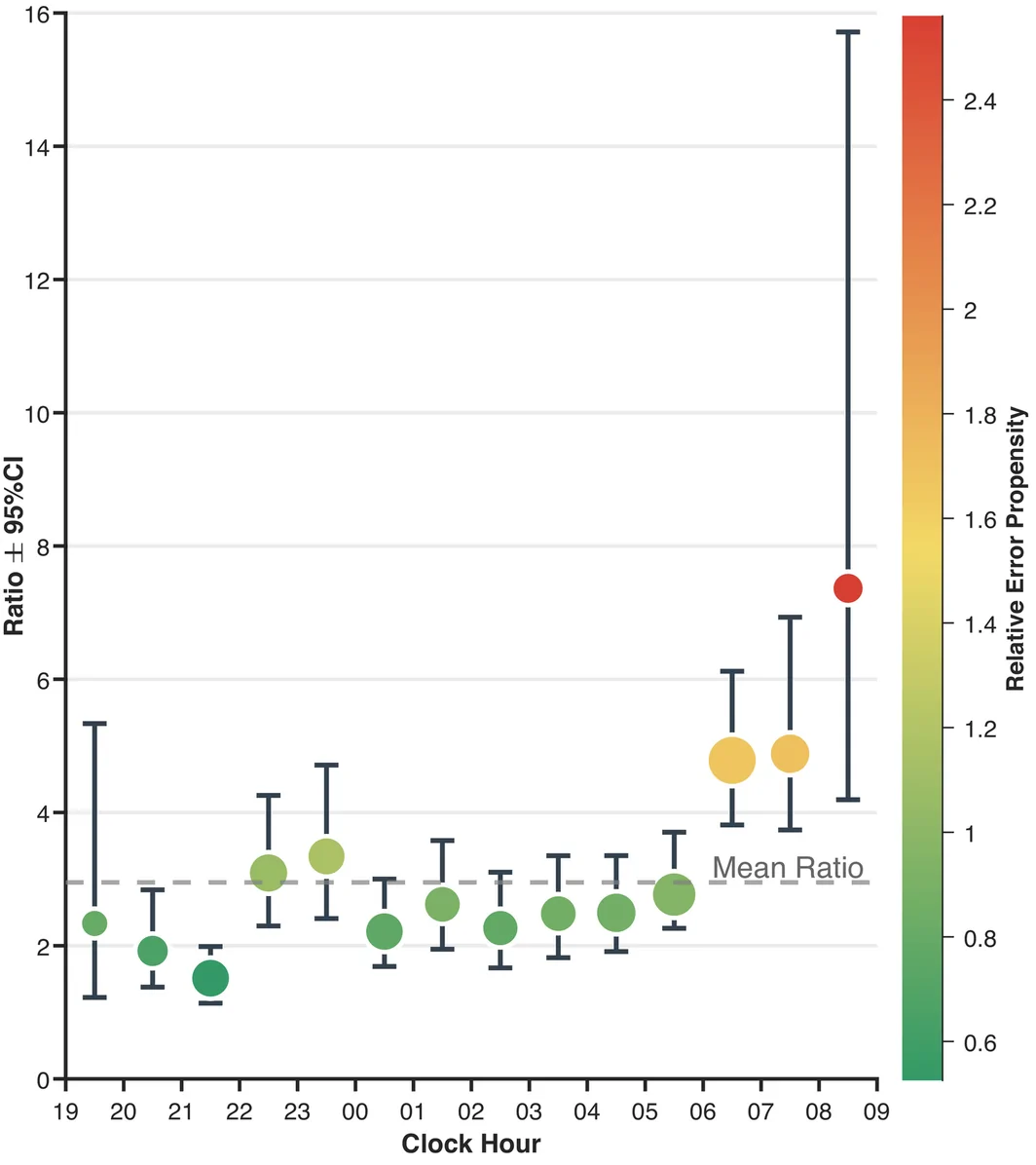
Hourly ratios of false‐to‐true alarms. Bubble size represents total alarm volume, indicating the statistical weight of each hour. Bubble color illustrates the relative error propensity, calculated as the hourly proportion of total false alarms divided by the hourly proportion of total true alarms; darker shades highlight hours where false alarms are disproportionately concentrated compared to true alarms. The horizontal dashed line represents the overall nightly mean ratio (~2.88). Hourly ratios appearing above this line indicate periods where false alarms are more frequent than the nightly average.

## 4 Discussion

Our findings suggest that the high frequency of false alarms in pediatric nocturnal seizure monitoring is not random, but instead reflects a time‐dependent phenomenon. While previous work has demonstrated that false alarms are broadly lower during overnight rest compared to active daytime periods [14], our findings expand on this by demonstrating that the performance of the NW system is contingent upon the specific timing within the nocturnal period, with a significant temporal cluster of false alarms in the early morning, coinciding with the period typically associated with wakefulness and morning arousal. Crucially, because the NW automatically suppresses alerts when the user leaves a recumbent position, this morning surge only reflects in‐bed detection of increased motoric or autonomic activity.

We noted another contrast in the early evening (19:00–22:00), which was characterized by relatively fewer false alarms and relatively more true alarms. We speculate that this may be due to a lower likelihood of arousals or awakenings causing false detections. Alternatively, this may reflect a higher propensity for seizure occurrence. Ultimately, our findings argue for exploring time‐ or state‐dependent detection thresholds to potentially reduce false alarms.

By adopting a population‐averaged GEE framework, we aimed to identify cohort‐level temporal patterns in NW performance rather than child‐specific trajectories. This approach enabled the identification of common time periods associated with increased false alarms but may have obscured individual temporal patterns that vary between children [15]. Although the GEE model accounts for the correlation of repeated alarms within the same child, it estimates population‐average effects rather than child‐specific differences. Furthermore, our cohort size and follow‐up duration were not designed to characterize individual periodicities reliably. Instead, our analysis identified cohort‐wide temporal hotspots, informing scalable and broadly applicable strategies to mitigate false alarms. Moreover, the selected 14‐h overnight monitoring window (19:00–09:00) may not have included all sleeping periods. Nonetheless, the frequency of events during the daytime hours (09:00–19:00) was insufficient for meaningful analysis.

The concentration of false alarms in the morning has direct practical implications; a disproportionate alarm burden during this window may contribute to caregiver fatigue during times of reduced vigilance. Conversely, relative robustness during early‐ and mid‐night hours indicates that NW performance is appropriate when 'noise' associated with wake periods is minimal. This suggests that the NW performs relatively well during deep nocturnal sleep.

While we are unaware of studies specifically evaluating the determinants of alarm fatigue among SDD users, the timing of false alarms is critical. Because many false alarms occur near typical awakening times, they may be less disruptive than those occurring during deep sleep. Not all false alarms can be attributed to non‐seizure‐related movements or arousals, as some may have been caused by minor seizures. We could not reliably differentiate between the two because we relied on video recordings without electroencephalography. This is a limitation, as it introduces physiological heterogeneity and complicates strategies to reduce false detections. It does not, however, impact the clinical relevance of our findings; most caregivers indicated that their primary goal is to be alerted to major, dangerous target seizures that require immediate, acute intervention [16]. Ultimately, user preferences for alarm sensitivity may vary between clinical subgroups [16] and should be systematically accounted for in future adaptive thresholding algorithms.

## 5 Conclusion

We demonstrated that false alarms generated by the NW system are dependent on the timing within the monitoring period. These alarms are notably clustered in the early morning, which might be explained by wakefulness and morning arousal. This identified temporal hotspot highlights a critical vulnerability in current detection algorithms that rely on static thresholds. By identifying these cohort‐wide performance trends, we argue for the development of dynamic detection models. Exploring time‐ or state‐dependent strategies may reduce the burden of false alarms. However, because true seizure events also populate these early morning hours, any modification to algorithm sensitivity carries an inherent clinical risk of missing genuine, potentially life‐threatening seizures. Consequently, implementing time‐ or state‐dependent thresholds will require future prospective validation.

## Author Contributions

Study conception and design: The study's overall concept and framework were jointly conceived and designed by M.S., A.T.B., and R.D.T., who together refined the research objectives, hypotheses, and approach. Data collection: A.T.B. and R.H.C.L. were responsible for gathering the data required for the study, including designing and implementing the data collection process, ensuring accuracy, and maintaining the integrity of the dataset. Analysis and interpretation of results: The analysis and interpretation of the data were jointly carried out all co‐authors. This included statistical evaluations, synthesis of findings, and drawing meaningful conclusions in the context of the study's aims. Draft manuscript preparation: M.S. took the lead in drafting the manuscript, organizing the findings, and presenting them in a coherent narrative that aligned with the study objectives and methods. Review and approval: All authors contributed to the critical review of the manuscript, ensuring the accuracy and completeness of the results and interpretations. Each author provided feedback and revisions, culminating in approval of the final version for publication.

## Funding

This work was supported by EpilepsieNL (2023–04) and the Anna Teding van Berkhout Stichting. The funders had no role in the study design, data collection, analysis, interpretation, or decision to submit for publication.

## Ethics Statement

Both trials were approved by the Medical Research Ethics Committee of the University Medical Centre Utrecht. We confirm that we have read the Journal's position on issues involved in ethical publication and affirm that this report is consistent with those guidelines.

## Consent

Written informed consent was obtained from the participants or assent from parents or legal guardians.

## Conflicts of Interest

Roland D. Thijs (R.D.T.): Reports lecture and consultancy fees from Medtronic, UCB, Angelini Pharma, Theravarance, Zogenix, Novartis, LivAssured, and Arvelle, as well as grants from Medtronic and NewLife Wearables. The other authors declare no conflicts of interest. The authors are part of the Dutch TeleEpilepsy Consortium; none of the authors have shares in LivAssured, the company developing the NightWatch device, nor have they received or will they receive any compensation referring to future sales of the NightWatch. LivAssured had no role in the study design, analysis, or decision to submit the manuscript.

## Data Availability

The data that support findings of this study are available from the corresponding author upon reasonable request.
